# Evidence-Based Role of Nutrients and Antioxidants for Chronic Pain Management in Musculoskeletal Frailty and Sarcopenia in Aging

**DOI:** 10.3390/geriatrics5010016

**Published:** 2020-03-06

**Authors:** Simone Perna, Tariq A. Alalwan, Salwa Al-Thawadi, Massimo Negro, Mauro Parimbelli, Giuseppe Cerullo, Clara Gasparri, Fabio Guerriero, Vittoria Infantino, Mariaconcetta Diana, Giuseppe D’Antona, Mariangela Rondanelli

**Affiliations:** 1 Department of Biology, College of Science, University of Bahrain, Sakhir Campus, Zallaq P.O. Box 32038, Kingdom of Bahrain; talalwan@uob.edu.bh (T.A.A.); salthawadi@uob.edu.bh (S.A.-T.); 2 CRIAMS-Sport Medicine Centre, 27058 Voghera, Italy; massimo.negro@unipv.it (M.N.); mauro.parimbelli@gmail.com (M.P.); gdantona@unipv.it (G.D.); 3 Department of Movement and Wellbeing Sciences, University of Naples “Parthenope”, 80133 Naples, Italy; giuseppe.cerullo@uniparthenope.it; 4 Endocrinology and Nutrition Unit, Azienda di Servizi alla Persona ‘‘Istituto Santa Margherita’’, University of Pavia, 27100 Pavia, Italy; clara.gasparri01@universitadipavia.it (C.G.); viriainfantino@hotmail.it (V.I.); 5 Department of Internal Medicine and Therapeutics, Section of Geriatrics, University of Pavia, 27100 Pavia, Italy; fabio_guerriero@yahoo.it; 6 Azienda di Servizi alla Persona, Istituto di Cura Santa Margherita of Pavia, 27100 Pavia, Italy; diana.mariaconcetta@gmail.com; 7 Department of Public Health, Experimental and Forensic Medicine, University of Pavia, 27100 Pavia, Italy; mariangela.rondanelli@unipv.it; 8 IRCCS Mondino Foundation, 27100 Pavia, Italy

**Keywords:** persistent pain, elderly, sarcopenia, antioxidants, nutrition

## Abstract

Musculoskeletal disorders in aging and pain are closely connected because of multiple mechanisms leading to loss of mobility and autonomy. Pain is predictive of diability and worsening frailty and the strength of this relationship increases with the severity of pain. This study presents a systematic review of randomized controlled trials, cross sectional studies, and observational studies based on treatment of pain in adults with musculoskeletal disorders using nutritional non-pharmacological (nutrients and antioxidants) interventions. The review found the efficiency of the following topics: (a) accession of the patient to a dietary counselling (e.g., daily recommended amount of protein—equivalent to at least of 1 g of protein per kilogram of body weight); (b) intake of glutamic acid-rich such as soy, egg, and cod and tryptophan-rich foods such as milk and peanuts—or taking quick-acting, free-form supplements; (c) supplementation of vitamin D and magnesium, if lacking; (d) weekly consumption of fish or supplements of omega-3 fatty acids; and (e) availability of botanicals, in particular curcumin and gingerol. These non-pharmacological interventions can help the pain therapist to create a personalized medicine (precision medicine), acting with the maximum efficacy and safety, and also reducing the dosage of analgesic drugs needed.

## 1. Introduction

Pain has been suggested to act as a stressor during aging and it accelerates functional and health status decline. Older adults suffering from pain are less physically active [1], experiences more comorbidities [2], and worse functional mobility [3] than older adults without pain. These adverse pain-related negative consequences may be responsible for the increase in the risk of developing sarcopenia and frailty, commonly observed in this age-group. Recent findings agree that pain is predictive of incident and worsening frailty [4].

The link between pain and the incidence of frailty rise with the intensity of pain in a dose-response modality [5]. In general, chronic and severe pain could affect several physiological systems, decreasing organ response to stressors and the ability to maintain homeostasis [6]. Pain and the pain-related adverse aspects may create a general state of vulnerability to stressors, which could lead older adults to increase their risk of developing or experiencing worsening frailty. The more pain a person suffers, the greater is the decline in physical activities, muscle mass/muscle strength and autonomy, and therefore the greater is the risk to develop sarcopenia and, subsequently, frailty. All these evidences could link pain to sarcopenia and frailty, typically in aging, getting them into a cyclic relationship similar to the well-known phenotype for frailty [6].

Clinicians face several challenges when treating pain (acute or chronic) in the elderly. Managing pain in older adults is a complex task due to the high prevalence of multiple comorbidities, polypharmacy, and, in general, socio-psychological vulnerability. Moreover, drugs-related adverse events (AEs) can be potentially harmful and hardly manageable in older adults (e.g., non-steroidal anti-inflammatory drugs and opiates AEs), even though drug prescriptions are generally individualized and tailored to patients’ health and social status [7]. Based on this, pain management in the elderly may be more effective when the intervention is based on a multidisciplinary method (e.g., psychological treatment, exercise program, complementary medicine, nutrition, and dietary supplementation) [8].

Of note, nutrition and nutraceuticals have proved to have a role in pain management and to help the patient overcome chronic pain, thereby improving the quality of life. Researchers have identified several nutritional components that may improve chronic pain syndromes through anti-oxidant and anti-inflammatory activities [9,10]. These novel properties of food and nutrients are very interesting and deserve attention from clinicians and researchers. In older adults, in particular those with sarcopenia and frailty who are more prone to drugs-related AEs, nutrition and nutraceuticals may play a relevant role and potentially assist in developing a therapeutic plan more effective to manage pain rather than exclusively based on the use of anti-inflammatory or pain reliever drugs. Hence, our aim is to review consistent literature and to perform a systematic review on the role of nutrients and nutraceuticals in sarcopenia and frailty aging-related pain (both acute and persistent) management.

## 2. Materials and Methods

The present systematic review was performed following the steps by Egger et al. [11]. Suitable for the systematic review were randomized controlled trials, cross sectional studies and observational studies which considered elderly with musculoskeletal pain and/or inflammation and musculoskeletal disorders. Search strategy was based on medical subject headings as follow: (musculoskeletal pain OR back pain OR pain management) AND (nutritional interventions OR antioxidant OR nutraceuticals) AND adults. Two hundred articles were retrieved in the initial search from “PubMed” and “Scopus”. Following removing articles with duplicate citations, 100 articles were screened based on their title and abstract. Forty-nine articles were identified as the most relevant articles with the purpose of this systematic review for the full text assessment. Selected collection of the data obtained is summarized in Table 1, Table 2 and Table 3.

## 3. Results

### 3.1. Proteins Rich in Essential Amino Acids

Elderly patients that have significantly lower skeletal muscle mass and strength are considered to be associated with chronic pain [12,13].

It is well recognized that adequate daily protein intake is required to preserve muscle mass and strength in older adults [14]. The source and quality of proteins is a key point underlined in recent studies that describe the essential amino acids (EAA) the most effective one in the control of muscle anabolism [15,16]. EAA regulate the protein synthesis [16] and leucine, in particular, promotes molecular events associated with muscle hypertrophy.

Leucine supplementation showed to overcome the anabolic resistance typically observed during aging, providing evidence that an increase of leucine consumption (i.e., 3–6 g) may play a key role to sustain muscle mass in elderly population [17]. Increasing evidence describe milk-derived whey proteins as an effective protein source to promote muscle protein synthesis and to stimulate muscle mass over time. Conversely, other data from middle-aged and older adults have shown that consumption of milk do not provide additional muscle benefits compared to resistance exercise alone [17].

A randomized-controlled trial showed that an increase in dietary protein (consumption of lean red meat three days per week) combined with progressive resistance training, enhance muscle mass, muscle size, and strength in community-dwelling older people [18]. Nevertheless, other findings showed that a modest increase in dietary protein intake (consumption of lean red meat two times per day) combined with progressive resistance training in a vitamin D-replete state has resulted in a greater increase in total body and regional lean tissue mass, muscle size and strength, and functional performance than with progressive resistance training alone in elderly women [19]. These data suggest as a moderate weekly consumption of lean red meat, properly balanced with fish (see below) and other alternative protein source rich in leucine (i.e., whey proteins), can be considered in a personalized diet plan to support muscle structure and function in order to prevent sarcopenia and its pain-related conditions.

### 3.2. Proteins rich in Glutamic Acid and Tryptophan

Researchers have identified many nutritional components that can improve disease-related persistent pain through proper antioxidant and anti-inflammatory activities. Endorphin, serotonin, and gamma-aminobutyric acid (GABA) appear to be the three primary neurotransmitters and pain modulators that are synthesized from amino acids.

Endorphin is a term used to identify a group of endogenous opioid neuropeptides that are produced by the central nervous system (CNS) and the pituitary gland that can powerfully reduce pain. Primary among them are the beta-endorphins and the enkephalins. These endogenous opioids are thousands of times stronger than morphine as pain relievers.

The effects of nutrition on the inflammatory pathway may represent the rational approach for an effective analgesic intervention. From this point of view, an increase of dietary protein intake can lead to a reduction in inflammation mediated by the increase in circulating IGF-I. Anti-inflammatory properties of IGF-I are based on a regulation network involving muscle-derived IL-6: high concentrations of IL-6 reduces IGF-I in serum and low IGF-I concentrations stimulates IL-6 release, suggesting that IL-6 can impair the effects of IGF-I on muscles [20].

By the 1980s, Seymour Ehrenpreis had observed that the D-phenylalanine endorphin has protective properties. Since then, several researchers have confirmed the positive properties of this amino acid in both acute and persistent pain management [21,22]. Authors have shown that the amino acid d-phenylalanine (DPA) reduces the activity of enzymes (in particular, carboxypeptidase A or endorphinase and enkephalinase) involved in the endorphin degradation pathway [23,24].

In clinical experience, it has been observed that pain relief occurs within ten minutes after the ingestion of as little as 500 mg of DPA, with a usual dose of 500–2000 mg of d-phenylalanine, two to four times a day, in patients with persistent pain [25].

In a vicious circle, muscle wasting is a well-known occurrence in chronic pain and post-operative patients. Remarkably, some studies have observed that both in patients after surgery and those with chronic pain, the consumption of 90–100 g of protein per day can prevent significant muscle-wasting and neurotransmitter level depletion [26,27].

Considering that endorphin, serotonin, and GABA are synthesized from amino acids present in high protein foods, it is important to recommend, through dietary counselling, to patients with pain to take the right amount of protein each day, equivalent to at least of 1 g of protein per kilogram of body weight, assuming both plant and animal proteins in each of the three main meals (breakfast, lunch and dinner).

In particular, protein foods rich in glutamic acid such as soy, egg, cod, and protein foods rich in tryptophan such as milk and dairy products and even peanuts are recommended. In the case that patients, for whatever reason, are unable to take the appropriate quantities, it is recommended to take proteins and amino acids in the form of quick-acting, free-form supplements.

### 3.3. Dietary Fatty Acids

Dietary fatty acids may affect muscle aging by a modulation of processes that involved inflammatory pathways, muscle anabolic and catabolic mechanisms. Saturated fatty acids, such as palmitic acid and stearic acid, promote inflammation responses in various cell types (monocytes, macrophages, and myocytes). Saturated fatty acids increase levels of inflammatory cytokines, such as tumor necrosis factor-α (TNF-α), which are involved in muscle protein breakdown. According to this pro-inflammatory pathway, saturated fatty acids could have a negative role on muscle health. On the other hand, omega-3 fatty acids have anti-inflammatory properties, and consequently, a positive effect on tissues metabolism [28]. Eicosapentaenoic acid (EPA) and docosahexaenoic acid (DHA) are long-chain polyunsaturated fatty acids (LC-PUFA, omega 3), particularly concentrated in fish oils and available in fish oil supplements. Both EPA and DHA, synthetized from α-linolenic acid, can decrease inflammatory response through a variety of mechanisms of action, acting as lipid mediators [28,29]. Based on this, current data are very promising and show that an adequate intake of omega-3 fatty acids may be useful in the control of inflammation-mediated pain in sarcopenic patients. The goal must be reached through increasing their intake of fish (4 times/week) or taking omega-3 PUFA supplements.

### 3.4. Magnesium

Dietary magnesium (Mg^++^) is shown to prevent age-related decrease of muscle mass and function (strength in particular), regulating well known mechanisms such as protein synthesis, ATP production, oxygen uptake, glycogen breakdown, fat oxidation, and electrolyte balance [30,31]. Furthermore, Mg^++^ could reduce the circulation of inflammatory cytokines, and in this regard a higher dietary intake of Mg^++^ correlates inversely with low circulating C-reactive protein levels with a positive impact on chronic low-grade age-related inflammation [32]. In conclusion, an appropriate dietary magnesium intake should be guaranteed, and in specific cases of low intake by the diet a Mg^++^ replacement with appropriate case-by-case supplementation may have a beneficial effect on ameliorating the pain associated with age-related loss of skeletal muscle mass and inflammation.

### 3.5. Vitamin D

It is well known that vitamin D plays a key role in bone homeostatis, autoimmune diseases, cell growth, inflammation or neuromuscular, and other immune functions. It is commonly accepted the correlation of Vitamin D status with osteomalacia, osteopenia, primary and secondary osteoporosis, but several studies have stressed the link with sarcopenia and with musculoskeletal pain.

Vitamin D is not only an essential hormone of bone metabolism, it also affects muscle strength, muscle size and neuro-muscolar performace. A decline of specific vitamin D receptors on muscle cells are directly associated with aging and with loss of muscle mass and function [33].

Moreover, several studies have also shown a relationship between musculoskeletal pain (e.g., low-back pain) and serum vitamin D level [34,35,36,37,38,39]. Among these studies, a cross-sectional study performed in Norway, including 572 patients with musculoskeletal pain, headaches or fatigue, concluded that more than half of all those subjects suffering from pain (58%) showed vitamin D levels < 50 nmol/L [40].

Moshfegh et al. showed that vitamin D deficiency is a contributor to diffuse a non-specific musculoskeletal pain [41]. A study that examined 150 patients with persistent, non-specific musculoskeletal pain of uncertain etiology found that as many as 96% had vitamin D deficiency [42].

According to other studies, pain could be linked to low levels of vitamin D because of the decreased bone mass, which predisposes people to osteoporotic fractures. In this regard, the post-menopausal estrogen deficiency leads to a bone mass loss, which predisposes to osteoporotic fractures but also to sarcopenia, that may be responsible for persistent low-back pain during aging [43].

The role of vitamin D, preventing loss of bone and loss of muscle, is also linked to inflammatory status. It has been supposed that a chronic reduction of muscle vitamin D receptors (VDRs) expression during skeletal muscle aging may compromise strength and functional capacity and may also be involved in an intramuscular inflammation processes, probably linked to non-genomic regulation mechanisms [44]. Vitamin D also suppresses the production of several pro-inflammatory cytokines detectable in blood serum, including IL-6 and TNF-α [45]. These inflammatory mechanisms may be relevant both in pain experience and in the pathology of sarcopenia.

In conclusion, data suggest a patho-physiological relevance of vitamin D and its deficiency for sarcopenia and pain. For this reason, it is mandatory to dose vitamin D blood-levels in older patients with these conditions. More trials are needed to determine if a personalized vitamin D replacement may have a beneficial effect on ameliorating musculoskeletal pain associated to sarcopenia and related frailty.

### 3.6. Botanical, Antioxidants and Nutraceutics

Ginger (Zingiber officinale) has been recognized as one of the most important plant with anti-inflammatory and analgesic properties. In adult subjects who ingested 2 g of ginger Vs placebo, the intensity of pain, typically associated with muscle damage, decreases followed the exercise. However, no statistical effects were generally registered after an acute fashion administration. In fact, only a small reduction in the increase of muscle pain was observed from the first to the second day following eccentric exercise, in participants who ingested ginger extracts during the 24 h after the exercise, and this effect was not increased by heat-treated ginger [46].

Curcumin, another plant-derived substance rich in antioxidant, is obtained from turmeric Curcuma longa. Curcumin, at the dose of 1 g two times per day (as the Phytosome delivery system, Meriva), 2.5 g twice daily, and 150 mg of lipid nanoparticle curcumin (Theracurmin, 1550 United Boulevard, Coquitlam, BC, Canada), respectively, can reduce the exercise-induced muscle damage expression with positive effects on muscle recovery, lower loss of maximal voluntary contraction, and lower increase of blood levels of creatine kinase [47,48].

In conclusion, taking the extracts of certain botanicals, especially curcumin and ginger, can be a valuable aide in controlling pain due to loss of muscle mass.

## 4. Discussion and Conclusions

This systematic review has shown that in subjects with age-related sarcopenia and frailty, specific nutritional and nutraceuticals interventions may play a relevant role in a long-term program of pain management. In particular, significant beneficial involvement of specific nutrients (i.e., proteins and amino acids, omega-3 fatty acids, magnesium and vitamin D), botanicals (curcumin and ginger) have been described.

The results of this study are useful to develop best-practice guidelines for a multidisciplinary management of pain that involve nutritional recommendation to support health professionals during the design of a personalized therapy, either in order to optimize therapeutic efficacy (i.e., improvement of the effect of a given intervention, for example drugs or surgical procedures) and safety (i.e., the prevention of harm to patients), or to reduce the dosage of analgesic drugs needed.

Treating both acute and persistent pain in older adults represents a complex task due to high prevalence of chronic co-morbidities, organ failures and age-related declining conditions. Moreover, older adults, especially those with sarcopenia and frailty, are more prone to experience analgesic-related AEs. A multimodal pain treatment—pharmacological and non-pharmacological approaches—is strongly needed in the elderly population because of better (effective and safe) outcomes. In this regard, a personalized approach including nutritional and lifestyle approach in older adults with sarcopenia and/or frailty condition is very important and helpful indeed.

Furthermore, based on the evidence, in our opinion, elderly people with chronic pain should undergo to a nutritional evaluation and controlled diet from the beginning of their care plan. The choice of foods and supplements to use must be tailored case-by-case in order to improve pain experience and clinical outcomes of analgesic treatment, finally resulting in considerable improvement of patient quality of life. From this current perspective, the potential benefits of nutrition and lifestyle changes during a personalized pain treatment program are highly promising.

## 5. Limitations of the Study

To the best of our knowledge, the present systematic review considers all the studies in the literature that have been analyzed for the inclusion and exclusion criteria considered. However, the number of references currently available on the subject is not yet large enough to allow greater precision in identifying, for the substances described, specific dosages to be recommended. This aspect represents the main limitation of our work and therefore requires further investigations.

## Figures and Tables

**Table 1:** Nutritional interventions.

First Author Year (Ref)	Number of Participant (M/F)	Age (y ± SD)	Setting	Inclusion Criteria	Exclusion Criteria	Supplement		Duration	Results	Conclusion
						Intervention	Control			
Katsanos et al., 2006 [49]	12/10	66.7 ± 2.0/66.5 ± 2.2	Aging Volunteers Registry	Elderly subjects, living independently with no limitations in ambulation	Unstable metabolic medical condition, hypertension, ECG-documented heart abnormalities, and vascular disease	2.8 g of leucine (41% Leu EAA)	1.7 g of leucine (26% Leu EAA)	2 days	FSR did not increase following ingestion of 26% Leu EAA (basal: 0.044 ± 0.003%/h; post-EAA: 0.049 ± 0.006%/h; *P* > 0.05) but did increase following ingestion of 41% Leu EAA	The results suggest that the EAA leucine has a unique role in the stimulation of muscle protein synthesis by EAAs in elderly humans
Daly et al., 2014 [20]	100 F	60–90	Self-care retirement villages	NR	Acute or terminal illness, unstable metabolic or cardiovascular disease, low-trauma fracture, type 1 diabetes, renal impairment, BMI > 40, the use of medication for muscle metabolism (corticosteroids or thyroxine), substantial weight loss	PRT with 160-g servings of cooked lean red meat/d; 6 d/wk for 4 mo	PRT with consuming ≥1 serving (∼75 g cooked) rice and/or pasta/d that provided ∼25–35 g carbo-hydrates	2 years	Statistical increase of 0.5-kg (95% CI: 0.1, 0.8-kg) in total body LTM in RT+Meat group compared to the CRT group; the proinflammatory cytokine IL-6 decreased significantly in the RT+Meat group after 4 month (P-group-by-time interaction < 0.05). An additional post hoc analysis showed that there was a 7.8% (95% CI: −15.7%, 0.0%) decrease in TNF-α in RT+Meat group after 4 months (*p* < 0.05)	A protein-enriched diet based on lean red meat is safe and effective for enhancing the effects of PRT on LTM and muscle strength and reducing circulating IL-6 concentrations in elderly women
Barbieri et al., 2003 [21]	222/304	65 ± 15; 66 ± 16	InCHIANTI Study	Population of InCHIANTI Study	Diabetes mellitus and major clinical cardiovascular diseases, people using drugs with interfere with IGF-I and IL-6 metabolism	NR	NR	2 years	Blood levels of IL-6 were positively correlated with age and BMI and negatively correlated with total power and handgrip strength. On the contrary, IGF-I’s blood concentration was negatively correlated with age and BMI and positively correlated with total power and handgrip	In older subjects with elevated levels of IL-6, the synthesis of production of IGF-I is diminished and the activity of the plasmatic IGF-I on muscle might be partially blunted
Welch et al., 2016 [50]	3519 F	34 to 83	Twins UK registry	NR	More than 10 answers about food items left blank or the ratio of estimated total energy intake to the estimated basal metabolic rate fell 2 SDs outside the mean ratio	Normal consume of dietary Mg in grip-strength group	Normal consume of Mg dietary in fat-free mass group	12 years	There was an inverse association between dietary Mg and hs-CRP in the adjusted model with a lower hs-CRP in the highest quintile of Mg intake (Q5) compared with Q1; an interquintile difference of 0.59 mg/L (p-trend = 0.011), equivalent to 28.9% of Q1	A higher dietary Mg intake was significantly associated in a beneficial direction with indices of skeletal muscle mass and leg explosive power, and also with circulating CRP concentrations The higher hs-CRP was negatively associated with lower indices of skeletal muscle mass
de Oliveira Otto et al., 2011 [32]	2466/2715	61.8 ± 10.3	The MESA population	The MESA study participants free of clinical CVD at baseline	Anti-inflammatory medications and suspected diabetes	Self-administered FFQ to assess usual food intake over the previous year	NR	NR	Dietary Mg intake was statistical inversely correlated with blood concentrations of tHcy but positively associated with fibrinogen. Participants in the highest quintile of Mg intake had 10% (95% CI: 7.0, 12.9) lower concentrations of plasma tHcy. On the contrary, participants in the highest quintile of Mg intake had ~3% (95% CI: 0.01, 4.7) higher plasma fibrinogen concentrations in comparison with those in the lowest quintile	The inverse association between Mg and tHcy is biologically plausible and consistent with the hypothesis that greater intake of nutrients with antioxidative/anti-inflammatory effects would be associated with lower levels of analytes reflecting inflammatory processes. Mg is an essential cofactor for several enzymes

NOTES: BMI, body massa index; CRP, c-reactive protein; CVD, Cardiovascular disease; EAA, essential amino acids; FFQ, food frequency questionnaire; FSR, fractional synthetic rate; IGF-I, insulin-like growth factor-I; IL-6, interleukin-6; Leu, leucine; LTM, lean tissue mass; MESA, multi-ethnic study of atherosclerosis; PRT, progressive resistance training; RT+Meat, resistance training plus lean red meat; tHcy, fasting total homocysteine. CI, confidence intervals; NR, reported; SD, standard deviation.

**Table 2:** Botanical and antioxidant compounds.

First AuthorYear (Ref)	Number of Participant (M/F)	Age (y ± SD)	Setting	Inclusion Criteria	Exclusion Criteria	Supplement		Duration	Results	Conclusion
						Intervention	Control			
Black et al., 2010 [46]	6/28	20	Campus of University of Georgia	Young volunteers	Performing moderate-to-high-intensity resistance training for biceps brachii muscle during the previous 9 months; taking prescription pain and/or psychiatric medication	2 g of ginger after exercise	Placebo	12 days	Pain-intensity ratings were significantly lower in the ginger group 24 h after eccentric exercise in both study 1 (Glass’s Δ = 0.78 SD, 25.3%, U = 85, *p* = 0.041) and study 2 (Δ = 0.57 SD, 22.5%, U = 127, *p* = 0.049).	Considerable evidence supports the biological plausibility of ginger possessing hypoalgesic effects.
Drobnic et al., 2014 [48]	20 M	38.1 ± 11.1	Sports Physiology Dept. of the O.T.C.	Healthy male, moderately active (regular cardio for at least 4 h per week), non-smoking volunteers	Treatment with anti-inflammatory/analgesic/antioxidant drugs, abnormal liver or renal function tests, active inflammatory or infectious or any kind of disease.	1g twice daily (corresponding to 200 mg curcumin twice a day) at breakfast and dinner	Placebo	4 days	Subjects in the curcumin group reported less pain in the lower limb as compared with subjects in the placebo group (total score: 23.3 ± 7.9 (17.2;29.4) vs. 30.6 ± 7.9 (24.9;36.2), *p* = 0.06)	The pain-relieving effect of curcumin supplementation could be mediated by a modulation of the inflammatory and oxidative responses to muscle injury.
Tanabe et al., 2015 [51]	14 M	23.5 ± 2.3	NR	Healthy, untrained young men not involved in any regular resistance training for at least 1 year before this study	No physical activities and assumption of anti-inflammatory drugs during the study period	150 mg of curcumin orally before and 12 h after each eccentric exercise bout	Placebo	4 days	Plasma IL-6 and TNF-α concentrations were not different between groups before exercise (IL-6 0.83 ± 0.22 vs. 0.73 ± 0.18 ng/mL, TNF-α 1.85 ± 0.74 vs. 1.63 ± 0.35 ng/mL, for curcumin and placebo, respectively). No change after eccentric exercise, and no differences between curcumin and placebo conditions	The study found that curcumin ingestion had no additive effects on blood markers of inflammation (IL-6 and TNF-α)

NOTES: IL-6, interleukin-6; TNF-α, tumor necrosis factor alpha. SD, standard deviation.

**Table 3:** Non-Pharmacological Treatments: Vitamin D.

First Author Year (Ref)	Number of Participant (M/F)	Age (y)	Setting	Inclusion Criteria	Exclusion Criteria	Supplement		Duration	Results	Conclusion
						Intervention	Control			
Knutsen et al., 2010 [35]	166/406	NR	Health center in which seven GPs serve 6200 patients (multi-ethnic area -north-eastern Oslo)	Analyzed Vitamin D levels in patients with headaches, fatigue, local or systemic muscle pain disease	Osteoporosis, injury, spinal herniation, rheumatic disease, and migraine	NR	NR	2 years	A total of 58% patients had low vitamin D levels (<50 nmol/L). Women had a higher degree of hypovitaminosis D than men (less than 30 nmol/L: *p* = 0.0005 and less than 25 nmol/L: *p* = 0.021). Headache was still significantly associated with hypovitaminosis D (*p* = 0.008, OR 2.6) after adjustment for gender, season, geographic region of origin, and age	The lowest levels of vitamin D were found among patients complaining of headaches
McCabe et al., 2016 [40]	3369 M	40–79	European Male Ageing Study	European Male Ageing Study With pain and vitamin D status	European Male Ageing Study without pain	Questions about lifestyle, including smoking and frequency of alcohol consumption and outdoor exercise. Pain level and localization. Serum levels of 25-(OH)D	NR	4.3 years	After adjustment for age and centre, compared to those in the upper quintile of 25-(OH) D (>36.3 ng/mL) those in the lowest quintile (<15.6 ng/mL) were more likely to develop CWP (OR = 2.32; 95% CI = 1.27–4.23	The men in the lowest quintile at baseline were more likely to develop CWP at follow-up than those in the upper quintile of serum 25 (OH) D, but this seem linked to the presence of harmful health factors, in particular obesity and depression. No statistical association was observed between 1,25 (OH)2D and the new occurrence of CWP (chronic widespread pain)
Plotnikoff and Quigley 2003 [42]	150 M/F	10–65	Community University Health Care Center (Minneapolis)	People with nonspecific musculoskeletal pain	NR	Vitamin D assay	NR	2 years	The prevalence of hypovitaminosis D was unexpectedly high in this population of nonelderly, non-house bound, primary care outpatients with persistent, nonspecific musculoskeletal pain refractory to standard pharmaceutical agents. Of all patients, 93% (140/150) had deficient levels of vitamin D (mean, 12.08 ng/mL; 95% confidence interval [CI], 11.18–12.99 ng/mL)	More than 90% of the patients in this study with persistent, nonspecific musculoskeletal pain were found to have deficient levels of 25-hydroxyvitamin D (this study also showed an unexpected disparity in hypovitaminosis D severity: younger patients had significantly lower 25-hydroxyvitamin D levels than did older patients)

NOTES: CWD, chronic widespread pain. CI, confidence intervals; NR, not reported.
